# Improvement in vision: a new goal for treatment of hereditary retinal degenerations

**DOI:** 10.1517/21678707.2015.1030393

**Published:** 2015-03-27

**Authors:** Samuel G Jacobson, Artur V Cideciyan, Gustavo D Aguirre, Alejandro J Roman, Alexander Sumaroka, William W Hauswirth, Krzysztof Palczewski

**Affiliations:** ^a^ ^1^University of Pennsylvania, Scheie Eye Institute, Perelman School of Medicine, Department of Ophthalmology, Philadelphia, PA, USAjacobsos@mail.med.upenn.edu; ^b^ ^2^University of Pennsylvania, School of Veterinary Medicine, Section of Ophthalmology, Philadelphia, PA, USA; ^c^ ^3^University of Florida, Department of Ophthalmology, Gainesville, FL, USA; ^d^ ^4^Case Western University, School of Medicine, Cleveland Center for Membrane and Structural Biology, Department of Pharmacology, Cleveland, OH, USA

**Keywords:** gene therapy, Leber congenital amaurosis, photoreceptors, retina, retinal pigment epithelium, retinoid cycle

## Abstract

***Introduction:*** Inherited retinal degenerations (IRDs) have long been considered untreatable and incurable. Recently, one form of early-onset autosomal recessive IRD, Leber congenital amaurosis (LCA) caused by mutations in *RPE65* (retinal pigment epithelium-specific protein 65 kDa) gene, has responded with some improvement of vision to gene augmentation therapy and oral retinoid administration. This early success now requires refinement of such therapeutics to fully realize the impact of these major scientific and clinical advances.

***Areas covered:*** Progress toward human therapy for *RPE65*-LCA is detailed from the understanding of molecular mechanisms to preclinical proof-of-concept research to clinical trials. Unexpected positive and complicating results in the patients receiving treatment are explained. Logical next steps to advance the clinical value of the therapeutics are suggested.

***Expert opinion:*** The first molecularly based early-phase therapies for an IRD are remarkably successful in that vision has improved and adverse events are mainly associated with surgical delivery to the subretinal space. Yet, there are features of the gene augmentation therapeutic response, such as slowed kinetics of night vision, lack of foveal cone function improvement and relentlessly progressive retinal degeneration despite therapy, that still require research attention.

## 1. Introduction

Inherited retinal degenerations (IRDs) are a group of blinding eye diseases now recognized to be caused by hundreds of different gene defects, mainly affecting photoreceptor cells and the retinal pigment epithelium 1, 2, 3. Prior to the era of gene discovery and known relationships of genes to specific diseases, clinicians specializing in IRDs tried to make sense out of the confusing array of symptoms and signs by subclassifying patients using Mendelian genetic type, age of disease onset, clinical features and retinal function. Clinical trials of treatment mainly sought to slow the natural history in groups of IRD patients using nutrient supplementation 4, 5.

The discovery of genetic causes of IRDs has continued at a steady pace over the last two decades with about 10 new genes discovered each year [6]. Knowing the causative genes and mutations provides a basis for understanding human IRDs. Pathophysiological mechanisms can now be postulated and, in rare instances, science-based treatments have been devised for these historically incurable diseases. Rather than a limited number of animal models for IRDs based only on phenotype, now there are naturally occurring or *de novo* generated models with gene defects that may mimic features of the genetically corresponding human diseases. With a few exceptions, consortia of basic science, animal and human retinal specialists have not worked together to understand a newly molecularly defined group of patients and how an animal model relates to that human disease. Some of the models move forward to be used for proof-of-concept studies; and human therapies have even been proposed based on results in animals that may or may not be faithful mimics of the human IRD.

About 15 years ago, advances in science and medicine came together to pave the path toward therapies for an early-onset autosomal recessive IRD. The form of Leber congenital amaurosis (LCA) that has now been treated is caused by that is caused by an abnormality in the visual (retinoid) cycle resulting from deficiency of RPE65. Animal models of RPE65 deficiency were available and proof-of-concept studies for two forms of therapy in young animals showed efficacy. This review summarizes the stepwise progress to treat humans with this form of LCA and suggests further directions to take now that early clinical trials of treatment have been successful. The data presented herein are mainly those of the authors. There are many recent reviews of retinal gene therapy or, specifically, gene augmentation in *RPE65*-LCA and these cover a spectrum of viewpoints of this rapidly advancing field 7, 8, 9, 10, 11, 12, 13.

## 2. RPE65 and the retinoid cycle

**Figure 1.**
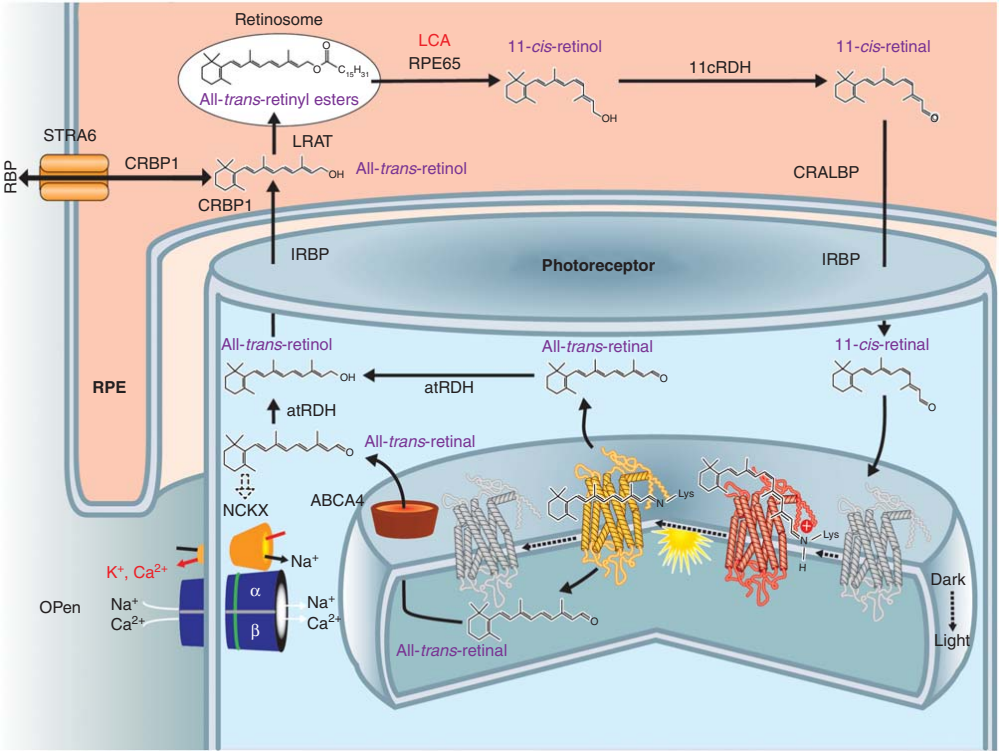
**RPE65 and the retinoid cycle.** Schematic of a retinal pigment epithelial (RPE) cell and a photoreceptor cell outer segment with the flow of retinoids within and between the two different cells. There are intracellular and extracellular retinoid-binding proteins (such as CRALBP, CRBP1, IRBP). The retinoid isomerase, RPE65 (top center of the figure), the retinoid isomerase, is deficient in a form of LCA. See text for more details.

Visual pigments in rod and cone photoreceptor cells detect light because they have the covalently linked light-sensitive chromophore, 11-*cis*-retinal. Light causes photoisomerization of this chromophore and its release as all-*trans*-retinal. Metabolic transformation of spent all-*trans*-retinal back to its light-sensitive form, 11-*cis*-retinal, is achieved by a series of transport and enzymatic processes termed the retinoid cycle. Combining recycled 11-*cis*-retinal with opsins to form visual pigments completes chromophore regeneration and permits phototransduction and visual perception to continue 14, 15, 16, 17, 18. One of the key enzymes of the retinoid cycle is retinoid isomerase, which is encoded by the *RPE65* gene (Figure 1). Deficiency of RPE65 leads to visual loss in human LCA. This visual disturbance is due not only to an inadequate supply of 11-*cis*-retinal but also to varying degrees of retinal degeneration 7, 19, 20, 21, 22.

The figure is reproduced with permission from [18]. CRALBP: Cellular retinaldehyde-binding protein; LCA: Leber congenital amaurosis.

The topic of the rod versus the cone visual cycle is worthy of comment. During the last two decades, a significant amount of biochemical evidence has indicated that Müller cells are involved in regeneration of cone visual pigments through a specific visual cycle pathway [23]. The key enzyme of this hypothetical pathway is thought to be the dihydroceramide desaturase-1 or Des-1 in conjunction with the key role of the cellular retinaldehyde-binding protein (CRALBP, encoded by *RLBP1*) in selection of the isomerization reaction products [24]. The Müller cells are also a source of 11-*cis*-retinal bound to CRALBP that is expressed in both RPE and Muller cells [25]. However, there is little genetic evidence from studies in humans and animals that provide proof of this pathway and its interplay with the ‘canonical’ RPE65-driven pathway.

## 3. Two preclinical experiments hold promise for treating human RPE65 deficiency

### 3.1 Remarkable improvement with oral 9-*cis*-retinoid in murine Rpe65 disease

**Figure 2.**
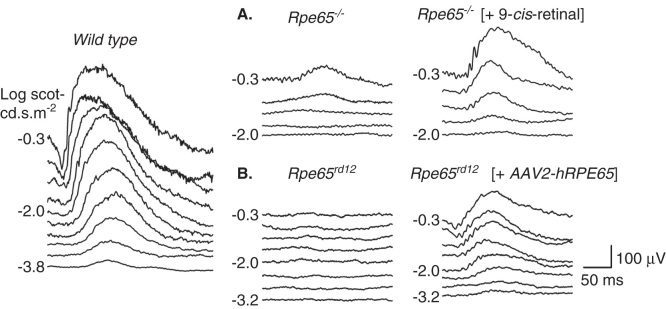
**Preclinical studies showing improvement in retinal function in Rpe65-mutant mice treated with oral 9-*cis*-retinoids** **(A) or subretinal gene delivery of AAV2-*Rpe65*** **(B)**. Left: Dark-adapted electroretinograms (ERGs) to increasing stimulus light intensity for a representative wild-type mouse. **(A)** ERG recordings in a representative 2-month-old *Rpe65* ^−/−^ mouse and in a 2.1-month-old *Rpe65* ^−/−^ mouse 48 h after oral 9-*cis*-retinal treatment. The treatment causes a lower stimulus threshold and larger amplitude ERGs. **(B)** ERG recordings comparing the untreated eye of a 2.9-month-old *Rpe65^rd12^* mouse and the contralateral eye treated with subretinal gene therapy. The treatment effect was a lowering of threshold and larger amplitude ERGs.

Given knowledge of the retinoid cycle, a pharmacological replacement therapy was devised and preclinical studies were performed in Rpe65-deficient mice and dogs 26, 27, 28, 29, 30, 31, 32. Studies in young mice with Rpe65 deficiency but without retinal degeneration demonstrated the feasibility of increasing visual pigment and retinal function by oral administration of a 9-*cis*-retinoid, thus bypassing the retinoid cycle blockade in this genetic disease (Figure 2A). The pathway for oral 9-*cis*-retinoid action is proposed to involve absorption in the intestine, storage in the liver, secretion into the blood and transport via a binding protein to the RPE [16]. 9-*cis*-retinal released from the RPE, possibly from storage sites composed of retinyl ester-containing lipid droplets, termed retinosomes 33, 34, combines with opsin to form isorhodopsin within photoreceptor cells. Isorhodopsin, investigated for more than half a century [35], is a photosensitive visual pigment proposed to be the source of residual function originating from rod photoreceptors in Rpe65-deficient mice [36] and the cause of improved vision after oral supplementation with 9-*cis*-retinoids 26, 27, 29, 30.

To explain further, opsin forms a stable pigment with 9-*cis*- 11-*cis*-retinal with two differences. First, the efficiency of photoisomerization for isorhodopsin is about one-third lower than that of rhodopsin. Second, the maximum absorption is shifted ∼ 7 – 10 nm to lower wavelengths (hypsochromic shift). Taking into consideration the range of human vision, these differences should be readily tolerated. Both isorhodopsin and rhodopsin when photoactivated form the same signaling intermediate termed metarhodopsin II [37], thus providing a sound molecular basis of this approach.

### 3.2 Remarkable improvement after subretinal gene augmentation in canine and murine Rpe65 disease

Surgically delivered gene augmentation therapy was also used to correct the abnormal retinoid cycle in canine and murine models of human *RPE65*-LCA. Subretinal gene therapy improved visual function in three *RPE65*-mutant dogs using a recombinant adeno-associated virus (AAV) vector-carrying wild-type canine *RPE65* cDNA [38]. This proof-of-concept experiment was confirmed and extended in many additional studies. Visual function improvements were recorded at retinal, subcortical and cortical levels, and there were concordant biochemical, morphological and immunohistochemical observations 7, 39, 40.

Subretinal gene therapy using AAV, adenovirus (Ad) and lentiviral vectors was also performed in the *Rpe65^−/−^* (knockout) mouse model as well as the naturally occurring *Rpe65^rd12^* mouse model. For the most part, there was improved retinal function (Figure 2B) with supporting immunohistochemistry, rhodopsin biochemistry and cortical activity studies [7].

## 4. Human RPE65 disease

Although there was promising preclinical evidence of efficacy with oral *cis*-retinoid and subretinal gene augmentation therapy in Rpe65-deficient animals, there were no studies specifically inquiring whether the disease expression in humans with *RPE65* mutations resembled that in the animal models. It was assumed to be similar enough. Young canine and murine models of Rpe65 deficiency, however, exhibited near normal photoreceptor structure despite severe rod and cone dysfunction that was reversed by the therapies. Other than in certain rare congenital stationary night or day blinding disorders, normal photoreceptor structure would be unusual in man. High-resolution optical coherence tomography (OCT) was used to quantify photoreceptor layer thickness in *RPE65*-LCA patients (ages 11 – 53) to define the relationship of retinal structure to vision [19]. Cone photoreceptor-rich central retina and rod-rich regions were specifically studied. Despite severely reduced cone vision, many *RPE65*-mutant retinas revealed a near-normal central microstructure. Absent rod vision was associated with a detectable but abnormally thinned photoreceptor cell layer. The human disease was thus not only a retinal dysfunction due to a biochemical blockade of the retinoid cycle but also a retinal degeneration. In contrast to other IRDs, however, *RPE65*-LCA patients showed greater photoreceptor nuclear layer integrity than predicted from their low level of vision [19]. In the mouse model, it was asked whether abnormally thinned *Rpe65*-mutant retina with photoreceptor loss, such as found in the human disease, could respond favorably to treatment. *Rpe65^−/−^* mice at advanced disease stages show photoreceptor cell loss and this represented a more faithful mimic of the human disease. When gene therapy or oral retinoid treatment were administered to these late-stage degeneration mice, retinal function did improve but only in animals with better preserved photoreceptor structure. To accomplish early-phase clinical trial goals of testing safety and efficacy of subretinal gene therapy, retinal locations with retained photoreceptors would need to be identified (with OCT) and targeted. Otherwise, the goals of the clinical trials would either not be achieved or left to a trial-and-error approach. Oral *cis*-retinoid therapy clinical trials would also be best conducted if patients were evaluated pre-enrollment using noninvasive OCT imaging. In other words, *RPE65*-LCA patients should be ‘staged’ for severity of degeneration prior to entry into clinical trials (see Section 7). Results of the trials would then be more interpretable.

Further studies of retinal photoreceptor structure in young patients with *RPE65*-LCA (ages 6 – 17) revealed that there was considerable interindividual variation and a simple relationship of age to severity of degeneration could not be assumed [21]. Summary maps of photoreceptor cell topography showed that superior-temporal and temporal pericentral retina was better preserved than other regions. Only more recently were natural history studies performed in the Rpe65 deficient dogs and mice and results used to decide when to administer treatment that would better approximate the human disease 41, 42.

## 5. Clinical trials of treatment

### 5.1 Subretinal gene augmentation therapy

**Figure 3.**
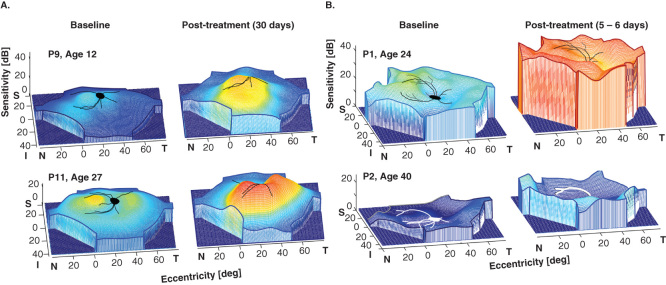
**Comparison of early efficacy post-treatment in subretinal gene therapy** **(A)** **and oral *cis*-retinoids** **(B)** **in *RPE65*-LCA.** Pseudo-three-dimensional representation of fully dark-adapted sensitivities across the visual field of the **(A)** gene-treated eye of two patients at baseline and 30 days post-treatment. P9 had a single subretinal injection in the superior retina; P11 had two subretinal injections, one in the superior retina and another in the nasal retina. **(B)** Baseline and 5 – 6 days after beginning a week-long course of oral *cis*-retinoids in one eye of two patients. Part B is reproduced with permission from [52].

Gene augmentation therapy for *RPE65*-LCA has been translated to the clinic and there are now multiple clinical trials worldwide with follow-up of at least 3 years showing safety and modest efficacy 7, 8, 9, 10, 11, 12, 13, 43, 44, 45, 46, 47. In the earliest versions of these trials, subretinal injection(s) of rAAV2-h*RPE65* was administered to the worse-functioning eye at various dose levels. Primary outcomes were systemic and ocular safety. Secondary outcomes assayed visual function with a variety of methods, including visual acuity, dark-adapted full-field sensitivity testing, visual fields, pupillometry and mobility performance. Both cone- and rod-photoreceptor-based vision improved in treated areas [46]. For extrafoveal cones, there were increases of up to 17 dB (50-fold); and for rods, there were gains of up to 48 dB (63,000-fold). A summary of the clinical trial (conducted by the authors) of 15 patients (age range, 11 – 30 years) showed there was no detectable systemic toxicity; any ocular adverse events were related to the retinal surgery [48]. Visual function improved in all patients to different degrees; and improvements were localized to treated areas. Cone and rod sensitivities increased significantly in study eyes but not in control eyes (Figure 3A). Minor visual acuity improvements were recorded in many study as well as control eyes. Major acuity improvements occurred in study eyes with the lowest entry acuities and parafoveal fixation loci treated with subretinal injections. Other patients with better foveal structure lost retinal thickness and acuity after subfoveal injections. It was concluded that *RPE65*-LCA gene therapy was safe and efficacious to the extrafoveal retina. To treat the fovea was of no benefit and carried some risk. There was no evidence of age-dependent effects [48].

#### 5.1.1 Unexpected results encountered to date

Detailed studies of the effects of gene therapy in patients with *RPE65*-LCA have produced several unexpected observations.

##### 5.1.1.1 Slow rod kinetics in treated retina

To assess what fraction of full visual potential was restored by gene therapy (taking into account the degenerative component), the relationship of the degree of light sensitivity to the level of remaining photoreceptors within the treatment area was determined. It was found that treatment could overcome nearly all the loss of light sensitivity resulting from the biochemical blockade component of *RPE65*-LCA. The reconstituted retinoid cycle, however, was not completely normal. Newly treated rods were remarkably slow to resensitize after light exposure and required 8 h or more to attain full sensitivity as compared to < 1 h for normal eyes. Cone sensitivity recovery time was rapid. These results demonstrated dramatic but imperfect recovery of rod and cone photoreceptor-based vision after *RPE65* gene therapy. In addition to theoretical interest in the basis of the slowed kinetics of rod adaptation, there is the practical implication that these patients need an extended time to dark adapt post-treatment or the visual gain in rod sensitivity would be underestimated. More concerning would be an apparent variability in magnitude of rod-mediated visual gain if insufficient (or different amounts of) dark adaptation was used to test for efficacy on different post-treatment assessments [46].

##### 5.1.1.2 Pseudo-fovea formation

A self-report by a patient about 1 year after gene therapy led to the observation that there could be late emergence of further visual gain in the treated eye. A pseudo-fovea developed and under certain conditions, the patient showed a preference to fixate at the treated retinal region (in the superior-temporal retina) rather than at the anatomical fovea. This suggested an experience-dependent plasticity of the adult visual system [49]. Further examples of this behavior have been documented subsequently and recently reported [50].

##### 5.1.1.3 Continued progression of retinal degeneration, independent of therapy

A key question about the effects of gene therapy on photoreceptor longevity was addressed recently [42]. As demonstrated previously, untreated *RPE65*-LCA patients show degeneration as well as dysfunction of photoreceptors even at the earliest ages. When examined serially over years, untreated *RPE65*-LCA outer photoreceptor nuclear layers (ONL) displayed progressive thinning. It has been expected, although unproven, that correction of the underlying cellular dysfunction by gene therapy could rescue the photoreceptors from degeneration. In treated *RPE65*-LCA, however, retinal degeneration also continued to progress despite the improved vision. This set of observations indicated that gene therapy in *RPE65*-LCA should not be considered as a permanent one-time treatment. An expanded view of the therapy is needed and ways to improve photoreceptor survival should be explored. Neuroprotective agents could be delivered independently to patients or in combination with gene augmentation and this could be tested experimentally in the canine model [42].

### 5.2 Oral retinoid therapy

Oral synthetic retinoid treatment (ClinicalTrials.gov Identifier NCT01014052) advanced to clinical trials in 7 *RPE65-*LCA patients and in another molecular subtype of LCA (lecithin retinol acyltransferase, *LRAT*) that also involves the retinoid cycle. Results of light-adapted metrics, such as visual acuity and kinetic perimetry, were reported to be improved following a week-long course of treatment with most returning to baseline by 2 years post-treatment. A minority of patients had longer-term persistence of improved acuity or visual field responses [51]. Considering the dramatic improvement in rod photopigment and rod physiology in the proof-of-concept studies of oral 9-*cis* retinoids in murine Rpe65 deficiency 19, 26, 27 it is of interest that results of dark-adapted perimetry were reported in two *RPE65*-LCA patients (ages 24 and 40) who received oral *cis*-retinoid [52]. Increases in dark-adapted sensitivity averaged 12 dB at 10 – 50% of loci. With extended dark adaptation, there were more dramatic increases in sensitivity of, on average, 16 – 19 dB over baseline at 40 – 83% of loci; individual loci could be as much as 24 – 36 dB increased (Figure 3B). The fact that extended dark adaptation led to further increases in rod sensitivity leads to the speculation that, like the slowed kinetics of rod adaptation in gene treated RPE65-deficient retina [46], the kinetics of rod adaptation after oral retinoid was also abnormally prolonged.

## 6. Comparison of the two treatments for *RPE65*-LCA

What are the differences between the results to date from surgically delivered gene augmentation therapy with AAV2-*RPE65* and the oral *cis*-retinoid treatment? Ocular gene therapy involves subretinal injection(s) of a certain volume of vector gene that is expected to transfect a localized region of the retina. Within a few days after this surgical procedure in one eye, there was evidence of an increase in rod vision in that eye limited to the region of subretinal treatment [48]. There is progressive retinal degeneration despite the improved vision [42], suggesting that vision would also eventually be impacted by the underlying cellular losses.

Oral *cis*-retinoid therapy is expected to affect retinal areas in both eyes, presumably those that retain sufficient photoreceptors and RPE to support the improved vision. Within days of therapy, there was evidence of an increase in rod vision across both eyes. The length of time that improvement in rod vision is sustained after oral *cis*-retinoid has not been reported; if predicted from rodent proof-of-concept experiments [29], it would be expected to diminish soon after dosing stops. It is also unknown whether the retinoid has any effect on the natural history of the progressive retinal degeneration in *RPE65*-LCA.

## 7. Next steps for treatment of *RPE65*-LCA

Ocular gene therapy of *RPE65*-LCA initially appeared deceptively simple with remarkable visual improvement occurring days to weeks after subretinal injection and apparent persistence of the positive effects for years. Treatment of the human disease (and later stages in animal models) has now been revealed to have limitations. Though augmenting wild-type RPE65 restored the retinoid cycle function (albeit not normally) and it improved rod and cone vision (but not foveal vision) in a measurable way, what was not measured, except in one of the clinical trials, was the degenerative component of this disease. If not quantified by serial optical imaging, the degenerative component would not have been noted to change until atrophy would become evident on ophthalmoscopic examination years to decades later. Clearly, the natural history of photoreceptor loss was independent of treatment [42]. The time course of visual loss that would be expected to eventually accompany the photoreceptor losses in treated and untreated retinal regions has not been reported.

Based on what is known to date of the effects of gene therapy in *RPE65*-LCA, it is obvious that the existing therapy is not fully adequate. What can be done now to advance the application of gene therapy to *RPE65*-LCA and other potentially treatable retinopathies?

### 7.1 Staging the disease

There is no strategy yet to define in detail patient candidacy for receiving gene therapy. The stage of the *RPE65*-LCA disease at the time of clinical presentation should serve as a guide to whether a subretinal injection procedure is an appropriate recommendation for that patient. Age of a patient has been assumed to be a major determinant of candidacy and benefit. By extrapolating from data of animal models of various IRDs, it has been assumed that an invariant decline in retinal function and photoreceptor structure with age is probably also what occurs in the population of patients with *RPE65*-LCA, despite a spectrum of different causative mutant alleles and diversity of genetic backgrounds. Are there data in humans to support this reductionist assumption? Individual untreated patients with *RPE65* mutations definitely show a decline in vision with age [22]. The kinetics of retinal degeneration in untreated human *RPE65*-mutant retina has also been investigated [42]. The conclusion from these studies is that there is no simple relationship between age and severity of disease when examining a population of patients with *RPE65* mutations. The onset, degree and spatial distribution of retinal degeneration across patients differ such that some young patients can have relatively severe degenerative disease while some older patients can have far better retinal preservation determined by careful imaging and visual functional analyses 21, 22. There are *in vitro* results for certain mutant *RPE65* alleles 53, 54, 55, but there are no data on the relationship of the many different mutations in the *RPE65* gene (and any modifier genes) to severity of *in vivo* disease expression. Considerable variation of severity of retinal degeneration exists in the first two decades of life. For patients above the age of 30 years, however, evidence of limited function and structure should provoke serious consideration of the risks versus the benefits of administering gene therapy 20, 21, 22.

Staging for severity of retinal degeneration is akin to performing biopsies as is commonly used to stage progressive nonocular diseases in order to make decisions about treating or type of treatment. To stage *RPE65*-LCA patients is within modern clinical capabilities. In *RPE65*-LCA, it can be accomplished noninvasively by *in vivo* imaging of the retina with OCT. Segmentation of these images and calculation of the average photoreceptor layer thickness across a wide retinal area would allow a patient’s retinal disease to be classified into one of possibly three stages (namely mild, moderate or severe). Patients without measurable photoreceptors would not be candidates for this therapy. For example, the average (and standard deviation) of photoreceptor layer thickness was measured across the central retinal region (30° × 30° square, 870 loci) in 12 *RPE65*-LCA patients and the patients were grouped as mild, moderate or severe (4 patients per group). In this sample, the statistically different averages were: 31 (6.7), 22.5 (6.1) and 11 (3.5) µm, respectively. Retrospective or prospective staging of all patients in ongoing clinical trials could be related to treatment efficacy by using some visual function criterion such as dark-adapted full-field sensitivity testing 56, 57. From such data a decision could be made about the frequency of treatment efficacy for each stage. In the future, it would be ideal and fair to present data to a patient about the chance of a successful outcome, rather than just a list of possible adverse events associated with retinal surgery.

Methods for rapidly testing interventions, such as in cancer treatment or prevention, are of strong interest to develop in the future, but have yet to be realized 58, 59. Based on the preliminary report about improvement in dark-adapted measures of vision using oral 9-*cis*-retinoid in two *RPE65*-LCA patients [52], it would seem worthwhile to consider this retinoid for use as a provocative test of the potential for efficacy in patients with *RPE65-*LCA before there is commitment to ocular surgery for gene therapy. This testing could be especially valuable for patients staged as ‘severe’ based on OCT average results. Some functional improvement from oral retinoid would suggest that a patient could be considered for gene therapy. A way to predetermine the value of gene therapy to a given patient would be a welcome addition to the therapeutic strategy for *RPE65*-LCA.

### 7.2 Disease management strategy

A long-term management strategy with gene therapy for *RPE65*-LCA also has not been proposed to date. All clinical trials began by treating one eye with a single subretinal injection. One clinical trial also treated two retinal locations in one eye in later cohorts to increase retinal coverage [48] and there is a report of second eye treatment administered years later [60]. Binocular strategies with single injections on separate days are also in progress (NCT00999609). Same eye retreatment has not been reported to date. Given a calculated stage of severity at first presentation, a photoreceptor life expectancy from time of initial OCT mapping can be determined using the delayed exponential model of the disease [42]. This prognosis should help define the long-term therapeutic strategy.

**Figure 4.**
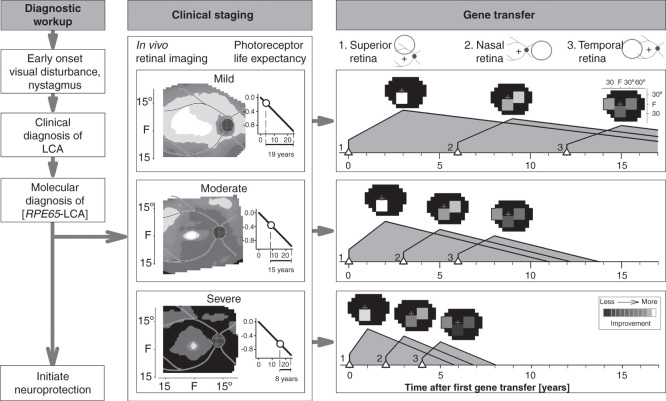
**An algorithm for gene therapy in *RPE65*-LCA.** Diagnostic workup is followed by clinical staging for severity of retinal degeneration. Photoreceptor life expectancy from time of initial mapping can then be calculated using the delayed exponential model of the disease. Simple or more complex strategies can then proceed with targeting of specific regions of retina proven to have photoreceptor integrity. Neuroprotection can be initiated as early as the diagnosis is made. See text for more details.

A sequence from clinical diagnosis to staging to treatment is illustrated (Figure 4). In the clinical staging column, there is a photoreceptor life expectancy graph. The horizontal axis is the time (years) since degeneration phase onset; ONL fraction remaining is plotted vertically in log units. The photoreceptor life expectancy for each stage (labeled below horizontal axis) is based on the patient’s average ONL fraction at time of imaging (marked by circle) until the ‘end-of-life’ criterion (−1 log unit ONL fraction, which is 10% of normal mean). A relatively simple gene transfer strategy with a series of single uniocular subretinal injections over years is shown. More complex strategies with two injections per eye and binocular treatments are possible. Initial administration targets the superior retina, as shown in the schematic of the fundus. Hypothetical outcomes are illustrated for each of three disease severities (see *Staging* above). Predicted visual improvement (at peak response) is shown as a square on a visual field map. After an interval determined by disease severity stage and the expected time to peak improvement, the nasal retina is treated. Treatment of the temporal retina could follow after a further interval, thereby providing a substantial total area of visual field that would otherwise be lost.

LCA: Leber congenital amaurosis.

In any future management strategy, it must be remembered that *RPE65*-LCA disease and gene therapy treatment effects are dynamic processes. Given the addition of a neuroprotective agent [42], the lifespan of photoreceptor cells could increase thus possibly lengthening the interval between follow-on treatments. Whether oral retinoid treatment becomes an alternative or a complement to gene therapy remains an important question.

### 7.3 Other treatment strategies

Nonviral gene therapy of Rpe65-deficient murine models has also been explored 61, 62, 63. Specifically, proof-of-concept experiments with subretinal DNA nanoparticles have been encouraging with long-term persistence of efficacy. Given further improvement of this method, concern over re-administration of AAV-mediated delivery could be eliminated if nonviral therapy was used. Problems with subretinal injection, however, would remain.

Staging of LCA patients with molecular evidence of disease-causing *RPE65* mutations will lead to identification of those with such severe photoreceptor and RPE loss (or who have some other exclusion criterion) that the gene-specific methods described above may not be appropriate. Such patients deserve consideration as possible candidates for other current methods being developed to improve severe vision loss [64]. For example, the development of a retinal prosthesis has a long history and there have been recent clinical trials; and further advances are expected. With evidence of extremely impaired vision, little or no measurable photoreceptors by OCT and presence of inner retinal laminae, this becomes one option 65, 66, 67, 68, 69. Another evolving method uses optogenetics to transform remaining inner retinal cells into light sensors [70]. Light-gated ion channels via a gene therapy approach are introduced into the residual inner retinal neurons 71, 72. Such optochemical and optochemical-genetic approaches have also shown proof-of-concept efficacy in large and small animal models of retinal degeneration [73].

Finally, the field of regenerative medicine is advancing such that stem cells of different origins can eventually become alternatives for improving vision in patients without sufficient photoreceptors to respond to current therapies 74, 75.

## 8. Expert opinion

The new challenge for clinicians will be to decide if or when novel therapies should be recommended and administered to specific IRD patients. As in all branches of medicine, treatment options create the need for decision-making. The most difficult decisions will require judgment based on knowledge of the topic(s). There will be pressure upon general ophthalmologists and retinal specialists who only rarely see IRD patients to be up-to-date (with the diseases, the natural history, the disease mechanisms) and be able to speak knowledgably about the latest therapies. Speaking from clinical experience will not be possible for many years. IRD patients tend to be well-informed about their diseases and will require more information from the physician than what they may have already gleaned from the popular media. There is now even more pressure than in past decades to administer therapy. Well-meaning patient organizations, for example, have fund-raised for decades to support basic and applied research, and have from the beginning promised clinical (not only basic scientific) progress. Their constituents have waited for these promises to be kept and there are accumulated feelings of obligation by organizations to announce and even promote treatments sometimes without the usual scientific scrutiny and complete knowledge of the outcomes and risks.

Will the workups and longer consultations needed to discuss novel therapies with the rare IRD patients fit into the active clinical schedules of ophthalmic practitioners? In the past, some of the busier practitioners found it expedient simply to provide a generally poor prognosis to their IRD patients. Ongoing needs of such patients including referrals for low vision and mobility training, career counseling, genetic counseling and even routine ophthalmic care to avoid complicating eye diseases were occasionally not pursued. Patients tended to learn from each other in support groups and at meetings of research-oriented foundations or associations to help the blind. The ophthalmic clinician will now have to practice an evolving form of personalized medicine for this group of orphan diseases.

For those who specialized in IRDs before the current novel therapeutics, personalized medicine has long been the only form of medicine practiced [76]. Now, there are simply more branches to the decision tree. A modified version of the four approaches to the practice of personalized medicine by Meyer [77] can be applied to *RPE65*-LCA. First is the assessment of an individual and the clinical diagnosis (from eye examination and electroretinography) and then molecular genetic testing. The latter demands a DNA sample and an infrastructure to submit it to a standardized approved laboratory and then the expertise in interpreting the result and explaining it to the patient/family 78, 79. Incidentally, there is no evidence to date that prenatal genetic diagnosis (e.g., in the case of a sibling with *RPE65*-LCA) is warranted, just as there is no evidence for administering gene therapy within the first years of life (see above). However, early diagnosis of *RPE65*-LCA remains a goal – mainly to be able to begin administration of a neuroprotective agent to alter the natural history [42]. Improvement of central cone function (given foveal cone structural integrity) is another plausible reason for early diagnosis and possible intervention. The latter could be a pharmaceutical agent or a gene therapy targeting an alternative cone visual cycle 15, 24 with an intravitreal vector delivery (but not subretinal surgery with its attendant risk of foveal cell loss). Second is to ‘increase diagnostic precision’ beyond molecular diagnosis by defining the phenotype with ‘prognostic and therapeutic implications’ [77]. Staging the retinal degenerative component of the disease using OCT is key to estimating the prognosis and deciding when to administer gene therapy (Figure 4). Third is ‘tailoring of treatment to the individual characteristics of each patient’ [77]. Even simple staging of the degree of degeneration would identify nonresponders. Improved tailoring of treatment to individuals could come from classifying patients by a predictable response to treatment derived from a consensus about how to measure efficacy in relationship to stage of degeneration in currently ongoing clinical trials (see above). The rapid move to higher phase trials has forced less rather than more information to be collected in the pursuit of ‘quality of life’ measurements. Focus should return to those details needed to understand a disease better and thereby improve the quality of its treatment. For example, gene therapy in *RPE65*-LCA has been popularized to an extent that it would seem to be in an advanced state, but some of the basics of the human disease, such as genotype–phenotype studies, have been bypassed in favor of a trial-and-error approach in the clinic and operating room. Clarifying whether patients with specific *RPE65* mutant alleles have different natural histories than others and require a specifically timed treatment strategy is essential. A starting population to study could be the group of *RPE65* patients with the same founder alleles in Israel to determine if there is a predictable natural history in this relatively homogeneous group [47]. A fourth tenet of personalized medicine from Meyer [77] is the ‘proper evaluation of objective and subjective clinical outcomes.’ OCT imaging, a feasible objective outcome for mechanism and safety, led to the observation that gene therapy did not alter the natural history of cell loss [42] and that foveal cell loss can accompany subfoveal injection 44, 48. The full-field sensitivity test after extended dark adaptation has offered the best assessment of outcome from these trials 46, 48, 57. The clinical utility of the therapy will not be improved until the impaired kinetics of dark adaptation are explained and corrected. The gain in night vision, one of the major improvements as a result of this therapy, is now constantly in jeopardy of being of no clinical utility unless the patients somehow learn to attend to their light history, a difficult way to live life in our well-lit modern world. Most reports by patients in the popular media are essentially proclaiming gains of extrafoveal cone vision.

Gene correction of IRDs, perhaps by a CRISPR-Cas9 or related approach, will advance and extend to other retinopathies 8, 10. The dual pathomechanism of *RPE65*-LCA disease, dysfunction and degeneration, however, is unlikely to be replicated in many other IRDs. Most IRDs will require natural history studies performed with quantitative outcomes for structure and function to try to detect negative changes in relatively short periods of time. Yet, there could be conditions in which photoreceptor outer segment lengths in residual cells would increase as a result of therapy and this could lead to some visual restoration. There are also retinal disorders that may be permanently cured by gene augmentation such as forms of congenital stationary night blindness that do not have a degenerative component [80].

In summary, treatment of IRDs seems to be at an inflection point, with early successes of gene therapy for *RPE65*-LCA deservedly infusing the field with optimism. However, careful clinical examination of these patients over time has revealed that simple, one-time gene therapy does not lead to a permanent therapeutic outcome and that there is a need to reexamine how clinical decisions are made relating to both initial treatment and follow-up options. This review has attempted to lay out a logical scaffold for dealing with these complex but ultimately approachable issues.

**Article highlights.**

- Inherited retinal degenerations (IRDs) are a group of blinding eye diseases that have long been considered untreatable and incurable.
- An autosomal recessive IRD called Leber congenital amaurosis (LCA), caused by mutations in the key retinoid cycle gene encoding RPE65, has recently been in clinical trials.
- Both gene augmentation therapy by subretinal injection and oral retinoid administration have led to some visual improvement in *RPE65*-LCA.
- There remain unresolved issues related to both therapeutic approaches that warrant attention before commercialization of the products.
- Most important is the issue of progressive photoreceptor cell loss (retinal degeneration) that continues in the treated regions even after vision-improving gene therapy.
- Efforts are needed to improve the quality and longevity of this novel treatment and thereby advance retinal gene augmentation therapy for *RPE65*-LCA as well as for other IRDs that will become candidates for this form of intervention.

This box summarizes key points contained in the article.
